# Measurements of True Leak Rates of MEMS Packages

**DOI:** 10.3390/s120303082

**Published:** 2012-03-06

**Authors:** Bongtae Han

**Affiliations:** CALCE Electronic Products and Systems Center, Department of Mechanical Engineering, University of Maryland, College Park, MD 20742, USA; E-Mail: bthan@umd.edu; Tel.: +1-301-405-5255; Fax: +1-301-314-9477

**Keywords:** MEMS package, hermeticity, helium mass spectrometer, true leak rate, gas conduction, gas diffusion, metallic seals, polymeric seals

## Abstract

Gas transport mechanisms that characterize the hermetic behavior of MEMS packages are fundamentally different depending upon which sealing materials are used in the packages. In metallic seals, gas transport occurs through a few nanoscale leak channels (*gas conduction*) that are produced randomly during the solder reflow process, while gas transport in polymeric seals occurs through the bulk material (*gas diffusion*). In this review article, the techniques to measure true leak rates of MEMS packages with the two sealing materials are described and discussed: a Helium mass spectrometer based technique for metallic sealing and a gas diffusion based model for polymeric sealing.

## 1. Introduction

Hermeticity of the MEMS package is a measure of the ability to maintain an acceptable level of stable and sometimes inert ambient in the cavity. It impacts device reliability and hence lifetime expectancy. Poor hermeticity can lead to ingress of contaminants, ambient gases and moisture, thereby causing performance degradation. Good hermeticity is essential for compliance with performance and reliability standards.

**Figure 1.**
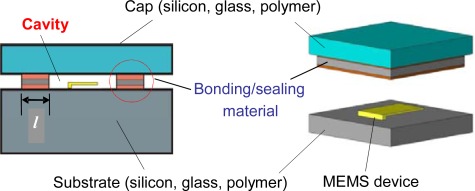
Schematic illustration of a MEMS package and the length of the leak channel, l.

A schematic of a typical MEMS package of interest is shown in Figure 1. As illustrated in the figure, the package comprises of a cap and a substrate bonded to each other such that they enclose a cavity between them which houses the MEMS device. Cavity volumes are typically less than 10^−3^ cm^3^.

The most commonly used sealing materials to provide hermeticity are low-melting point eutectics such as AuSn [1], AuSi [2] and other tin based alloys [3]. More recently, polymers have gained widespread acceptance due to several advantages that they offer [4]; they include lower processing temperatures, compatibility with integrated circuit wafers and the ability to join practically any kind of wafer materials [5]. In addition, polymer wafer bonding does not require special wafer surface treatments such as planarization and excessive cleaning since structures and particles on the wafer surfaces can be tolerated and compensated to some extent by the polymer adhesive [5]. Examples of polymeric seals include benzocyclobutene (BCB), parylene, polyimides and negative photoresists [6,7].

It is important to note that gas transport mechanisms in the two sealing materials are completely different. The helium fine leak test [8,9] is used to illustrate the difference. In the test, the package is first subjected to pressurized helium and then a helium mass spectrometer measures the rate at which helium leaks out while the package is subjected to a vacuum.

**Figure 2.**
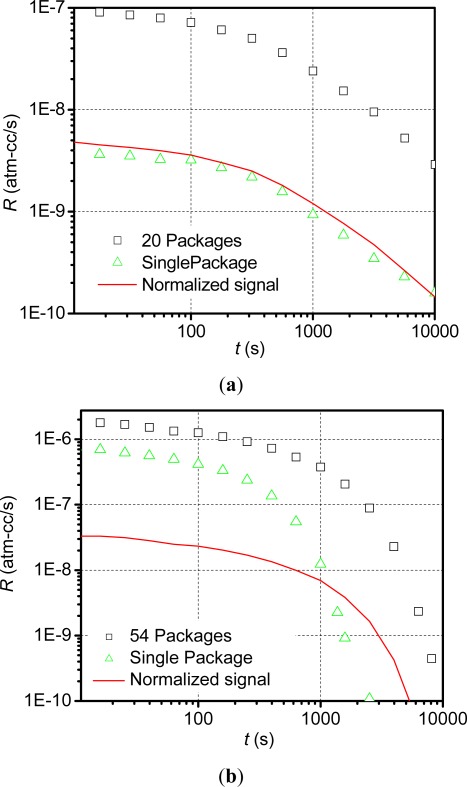
Helium leak test signals for (**a**) polymer-sealed packages (*V_cavity_* = 3.1 × 10^−4^ cm^3^) and (**b**) metal-sealed packages (*V_cavity_* = 2.156 × 10^−4^ cm^3^). Test parameters include a bombing pressure of 4 atm (gage), a bombing time of 6 h and a dwell time of 10 min.

Figure 2 shows helium fine leak test results obtained from two separate tests with polymer-sealed packages (*V_cavity_* = 3.1 × 10^−4^ cm^3^) and metal-sealed packages (*V_cavity_* = 2.16 × 10^−4^ cm^3^). The test conditions include a bombing time of 6 hours at 4 atm (gauge) and a dwell time of 10 minutes. In the experiment, individual packages as well as batches containing multiple identical packages were tested; in the batch tests 20 polymer-sealed packages and 54 metal-sealed were used. The signals obtained from the batch tests and single package tests are shown in Figure 2(a,b) for polymer-sealed and metal-sealed packages, respectively. The batch test signals normalized by the number of packages in the batches are also plotted for comparison.

The normalized signal of the polymer-sealed packages is similar to that of the single package signal (Figure 2(a)), which indicates that the polymer-sealed packages produce virtually the same signal when tested individually. This was confirmed by additional tests of individual packages used in the batch test. On the other hand, the normalized signal of the metal-sealed packages is much lower than that of the single package signal (Figure 2(b)), which indicates that the average signal of the batch does not represent the leak behavior of a single package. Additional tests confirmed that only five out of the 54 packages used in the batch test were leaky and these packages produce unique apparent leak rate profiles [10].

The above results clearly indicate the different gas transport mechanisms exist in the two packages. In metallic seals, gas transport occurs through a few nanoscale leak channels (*gas conduction*) that are produced randomly during the solder reflow process, and thus the leak rate depends on the gas molar mass and the geometry of the channel (diameter and length). On the other hand, gas transport in polymeric seals occurs through the bulk material (*gas diffusion*), and thus the leak rate depends on the gas diffusion properties (diffusivity and solubility) and the structure of polymer seals.

In this review article, the techniques to measure true leak rates of MEMS packages with the two sealing materials are described and discussed: a helium mass spectrometer based technique for metallic sealing and a gas diffusion based model for polymeric sealing [10–17]. Much of the manuscript is excerpted from [10,15] with the permission of the IEEE Intellectual Property Right Office through RightsLink.

## 2. Governing Equation

In both molecular gas conduction and gas diffusion, the gas flux can be described by the gas conductance equation in a general form as [18]:

(1) J=FΔp

where *J* is the gas mass flux (kg/m^2^sec), *F* is the gas conductance (sec/m), Δ*p* is the gas pressure differential (Pa). In the case of gas conduction, the gas conductance is derived from the kinetic theory of gases while it is determined from Fick’s first law in the case of gas diffusion. These expressions are [18]”

(2) $$F=\frac{d_{\mathrm{tube}}}{3L}\sqrt{\frac{8}{\pi {\mathrm{MR}}_{0}T}}\;\text{for gas conduction}$$

(3) $$F=\frac{P}{L}\;\text{for gas diffusion}$$

where *d_tube_* is the diameter of a nanoscale leak channel (m), *L* is the conduction or diffusion path length (m), *M* is the gas molar mass (kg/mol), *R*_0_ is the universal gas constant (8.3145 J/molK), *T* is the temperature (K) and *P* is the permeability of the gas (s). Although the two mechanisms are described by the same form of equations, there are two fundamental differences between them with regards to the geometry of gas transport paths and the time required for pressure gradient development inside the transport paths.

### 2.1. Gas Conduction for Metallic Seals

In gas conduction, gas molecules travel through a nanoscale channel and thus can be regarded as a Cartesian 1-D flow problem. The pressure gradient inside the flow channel is developed almost instantaneously, and transient effects are negligible. Thus, the gas transport can be predicted by simply considering the conduction equation (Equation (2)) with appropriate boundary conditions at both ends of the channel.

Conductance can be expressed in terms of the channel dimensions, fluid properties and ambient conditions. The exact expression depends on the nature of the flow regime. Gas flow is divided into three regimes based on the nature of the flow as determined by the ratio of the characteristic dimension of the leak channel (the radius of the circular cross section, *a*) and the mean free path, *mfp*, of the gas which is defined as the average distance traveled by the molecules of the gas between successive collisions. The mean free path is mathematically expressed in cgs units as:

(4) $$\mathrm{mfp}=\frac{\mathrm{kT}}{\sqrt{2}\pi p\sigma^{2}}$$

where *K* is the Boltzman constant, *σ* is the diameter of the molecule (Helium: 2.2 × 10^−8^ cm, Air: 3.7 × 10^−8^ cm [19]), *T* is the absolute temperature and *p* is the pressure of the chamber in which the gas is enclosed. In determining the *mfp* of a gas flowing through a leak channel, the average pressure, $p_{a}\left(=\frac{p_{u}+p_{d}}{2}\right)$, should be used in Equation (1).

The three flow regimes [18] include: (1) molecular flow regime where the mean free path of the gas molecules is large compared to the characteristic dimension of the leak channel and the rate of flow is limited by collisions of the molecules with the walls of the leak channel; (2) viscous flow regime where the mean free path of gas molecules is small compared to the characteristic dimension of the leak channel and the rate of flow is limited by intermolecular collisions; and (3) transition flow regime where the flow characteristics are determined by both intermolecular collisions as well as collisions between the molecules and the walls.

Analytical expressions for conductance in the molecular and viscous regimes are available in the literature [18]. Knudsen experimentally measured the conductance of a cylindrical leak channel and developed a semi-empirical equation for the total conductance, *F* that can be used in all flow regimes as [20]:

(5) $$F_{T}=F_{v}+{\mathrm{ZF}}_{m}=F_{m}\left(\frac{F_{v}}{F_{m}}+Z\right)\;\text{where}\;Z=\left(\frac{1+2.507\frac{a}{\mathrm{mfp}}}{1+3.095\frac{a}{\mathrm{mfp}}}\right)$$

where *F_T_* is the total conductance; *F_m_* is the conductance in the molecular regime; and *F_v_* is the conductance in the viscous regime,

### 2.2. Gas Diffusion for Polymer Seals

In contrast to gas conduction, gas diffusion takes place through the entire sealing area. Multi-dimensional modeling is necessary to account for the actual sealing layer structure. In addition, the gas pressure gradient inside the sealing material develops very slowly (usually on the order of hours to days). The conductance equation based on Fick’s first law cannot model such a slow pressure gradient development and hence Fick’s second law has to be considered.

Fick’s second law is derived from the principle of mass continuity for an infinitesimal volume as:

(6) $$\frac{\partial C}{\partial t}=\nabla \cdot (D\nabla C)$$

where *C* is the gas concentration (kg/m^3^), ∇ is the gradient operator and *D* is the gas diffusivity (m^2^/sec). Using the linear Henry’s law (*C* = *Sp*), Equation (6) can be rewritten as:

(7) $$\frac{\partial (\mathrm{Sp})}{\partial t}=\nabla \cdot [D\nabla (\mathrm{Sp})]$$

where *S* is the solubility (s^2^/m^2^) and *p* is the gas pressure (Pa). In an isothermal problem, the above equation can be simplified as:

(8) $$\frac{\partial p}{\partial t}=D\nabla^{2}p$$

The permeability *P* is defined as the product of solubility and diffusivity (*P* = *DS*). As shown in Equations (6) and (7), the permeability characterizes the steady-state gas flux through a polymer, thus can be measured from a gas transmission test. On the other hand, the diffusivity governs the transient gas transport inside the polymer, thus can be determined from a transient gas absorption/desorption/transmission test [18].

### Axisymmetric Formulation

The cavity of a typical MEMS package has a rectangular (or square) shape. Many parameters are required to define the structure of the cavity and the surrounding seal, and hence the rectangular shape is not most ideal for a parametric study. In this study we consider an axisymmetric model, which is much more effective for the parametric study. Although simplified, the axisymmetric model effectively represents the gas diffusion behavior of the actual cavity structure.

**Figure 3.**
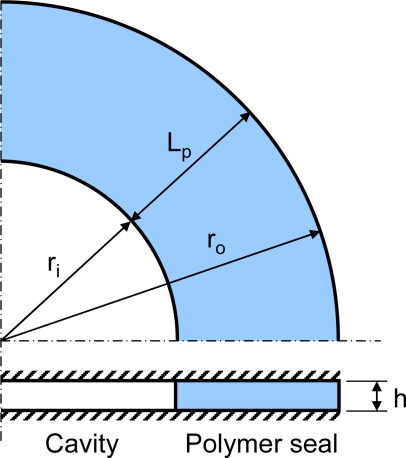
Schematic diagram of the geometry of 1-D axisymmetric case (the top and bottom surfaces are adiabatic).

An axisymmetric model is formulated to illustrate the transient boundary conditions. Its normalized form will be utilized later for an extensive parametric study. A schematic diagram of the axisymmetric model is illustrated in Figure 3. The axisymmetric form of Equation (8) can be expressed as:

(9) $$\frac{\partial p}{\partial t}=D\nabla^{2}\;p$$

and the boundary and initial conditions are:

(10) $$p(r_{o},\;t)=p_{a},\;\;p(r_{i},\;t)=p_{c},\;\text{and}\;p(r,0)=0$$

where *p_a_* and *p_c_* are the ambient and cavity pressure, respectively. The cavity pressure change during each time step (Δ*t*) can be calculated as:

(11) $$p_{c}(t+\Delta t)=p_{c}\;(t)-\frac{A_{i}\;R_{0}\;T}{{\mathrm{MV}}_{c}}\int_{\Delta t}J_{r=r_{i}}\;(t)\mathrm{dt}$$

where *A_i_* is the inner surface area (= 2*πr_i_h*), *M* is the gas molar mass (kg/mol) and *V_c_* is the cavity volume $\left(=\pi r_{i}^{2}h\right)$.

## 3. True Leakage Measurement of Metal-Sealed Package [10]

The helium mass spectrometer based leak testing has been widely used in the industry for fine leak detection [8,9]. In the helium fine leak test, the package is subjected to pressurized helium and then transferred to a helium mass spectrometer. The spectrometer measures the rate at which helium leaks out while the package is subjected to a vacuum.

The output of the spectrometer is the measured leak rate (*R*), which is defined as the leak rate of a given package as measured under specified test conditions [21]. The measured leak rate is also referred to as “*apparent leak rate*”, which decreases as a function of time. In practice only the *initial* apparent leak rate (*R_i_*), *i.e.*, the measured leak rate at the instant the spectrometer is switched on, is used as a measure of hermeticity [21].

The equivalent standard leak rate (*L_a_*) of a package is defined as the leak rate when the high-pressure side is at 1 atmosphere (760 mm Hg absolute) and the low-pressure side is at a pressure of less than 1 mm Hg absolute (*i.e.*, ≈vacuum) [21]. The equivalent standard leak rate is also referred to as “*true leak rate*”. The *true* leak rate (*L_a_*) is the characteristic of the package and is only a function of leak opening geometries, while the *initial* apparent leak rate (*R_i_*) is a function of the test parameters, the true leak rate and the specimen volume (*V*).

For relatively large packages, there exists one-to-one correspondence between the initial apparent leak rate and the true leak rate in the fine leak domain (less than 10^−4^ atm-cm^3^/s). As the package volume becomes smaller (less than 10^−3^ cm^3^), however, the one-to-one correspondence vanishes [11].

The consequence of this loss of one-to-one correspondence is that the initial apparent leak rate no longer carries quantitative meaning; for example, a package with a higher true leak rate (*i.e.*, poor hermeticity) can produce a lower apparent leak rate signal than a package with a lower true leak rate (*i.e.*, good hermeticity), thereby leading to erroneous interpretations of hermetic quality.

To cope with the problem, a method to extract the true leak rate using the Helium mass spectrometer has been developed. The method utilizes the complete profile of the apparent leak rate collected by the mass spectrometer and determines the true leak rate by performing a non-linear regression analysis. The theoretical limit of true leak rates that can be measured by the fine leak test was studied previously [11,12]. The theory and the procedure to extract the true leak rates in this measurable range will be presented in the following sections.

### 3.1. Procedure of Modified Helium Fine Leak Test

The first step comprises of “bombing” the specimen with helium, *i.e.*, subjecting it to helium pressurized at the bombing pressure, *P_b_*, for the bombing period, *t_b_*, and then transferring it to a helium mass spectrometer where a vacuum is pulled to measure the rate at which helium leaks out. It should be noted that in this measurement procedure there is “*dwell time*”, *t_dwell_*, between the instant the specimen is taken out of the bombing chamber and the instant the spectrometer is switched on, during which some of the helium escapes from the package.

Ideally the spectrometer should measure only the helium leaking out of the package, *i.e.*, the *actual* signal. In practice, however, a small amount of helium present in ambient air contributes to the signal in the form of noise [11,12]. This extra signal is called the “*zero signal*”. The zero signal becomes negligible as soon as the air present inside the test chamber of the mass spectrometer is drawn out. This short duration will be referred to as “*zero signal time*”, *t_zero_*.

For quantitative characterization of the true leak rate, stable apparent leak rate data must be utilized for consistency and accuracy. Therefore, the data to be used for a subsequent regression analysis should be taken only after the zero signal becomes negligible. We introduce a new parameter called *“preprocessing time”*, which is the sum of the dwell time, *t_dwell_*, and the zero signal time, *t_zero_*. Physically, the preprocessing time, *t_p_*, is the time that elapses from the instant when the specimen is taken out of the bombing chamber to the instant that collection of useful leak rate data is started.

### 3.2. Mathematical Formulation

An approach similar to the one outlined in reference [22] is adopted to model the modified helium leak test. In the analyses the initial pressure of the package cavity is assumed to be zero (at vacuum). It is also assumed that the leak path is a single cylindrical channel with radius, *a*, and length *l*.

The test is divided into three phases, viz. bombing, preprocessing, and measurement phases. For the purpose of mathematical modeling, the preprocessing phase is further divided into two sub-phases, the dwell phase and the zero signal phase, since the downstream pressure of each sub-phase is different (1 atm and vacuum for the dwell and zero signal phases, respectively). In each phase, the ratio between *a* and *mfp* (*a/mfp*) is continuously calculated to update the value of conductance using Equation (5). The conductance is used to determine outflow/inflow of gas and thereby calculate changes in the internal pressure.

#### 3.2.1. Bombing

During the bombing phase, the upstream pressure, *P_u_*, is equal to the bombing pressure, *P_b_*. The internal pressure of the cavity, *P_i_*(*t*), increases steadily and reaches a constant value of *P_i_*(*t_b_*) at *t* = *t_b_*, which can be expressed as:

(12) $$P_{i}(t_{b})=P_{i}(0)+\int_{0}^{t_{b}}\frac{R(t)}{V}\mathrm{dt};\;\;\;R(t)=F(t)\;(P_{b}-P_{i}(t))\;\text{for}\;0<t<t_{b}$$

where *V* is the cavity volume, and *R*(*t*) and *F*(*t*) are the apparent leak rate and the total conductance at any given instant during bombing.

#### 3.2.2. Dwell

In this phase the internal cavity pressure is the upstream pressure, *P_u_*, and it decreases steadily as helium leaks out of the package. The initial value of *P_u_* is equal to the final cavity pressure calculated at the end of the bombing phase, *i.e.*, *P_i_*(*t_b_*), and the downstream pressure, *P_d_*, is equal to 1 atm. The final cavity pressure after the dwell time can be calculated using the following equation:

(13) $$P_{i}(t_{b}+t_{\mathrm{dwell}})=P_{i}(t_{b})-\int_{t_{b}}^{t_{b}+t_{\mathrm{dwell}}}\frac{R(t)}{V}\mathrm{dt};\;\;\;R(t)=F(t)\;(P_{i}(t)-1)\;\text{for}\;t_{b}<t<t_{b}+t_{\mathrm{dwell}}$$

#### 3.2.3. Zero Signal Time

The internal cavity pressure is still the upstream pressure, *P_u_*. The initial value of *P_u_* is equal to the final cavity pressure calculated at the end of the dwell phase, *P_i_*(*t_b_* + *t_dwell_*) and the downstream pressure, *P_d_*, is equal to 0 (vacuum). The final cavity pressure after the zero signal time can be calculated using the following equation:

(14) $$P_{i}(t_{b}+t_{p})=P_{i}(t_{b}+t_{\mathrm{dwell}})-\int_{t_{b}+t_{\mathrm{dwell}}}^{t_{b}+t_{p}}\frac{R(t)}{V}\mathrm{dt};\;\;\;R(t)=F(t)\;(P_{i}(t)-0)\;\text{for}\;t_{b}+t_{\mathrm{dwell}}<t<t_{b}+t_{p}$$

#### 3.2.5. Measurement Phase

The internal cavity pressure is the upstream pressure, *P_u_*. The initial internal cavity pressure in the measurement phase is equal to the final cavity pressure calculated at the end of the preprocessing, *P_i_*(*t_b_* + *t_p_*). The internal cavity pressure at any time, *t*, *i.e.*, the instantaneous value of upstream pressure can be calculated using the following equation:

(15) $$P_{i}(t)=P_{i}(t_{b}+t_{p})+\int_{t_{b}+t_{\mathrm{dwell}}}^{t_{b}+t_{p}}\frac{R(t)}{V}\mathrm{dt};\;\;\;R(t)=F(t)\;(P_{i}(t)-0)\;\text{for}\;t>t_{b}+t_{p}$$

### 3.3. Determination of True Leak Rate from Apparent Leak Rate

The task of inferring *L_a_* from *R(t)* is to calculate the value of *L_a_* inversely from the apparent leak rate profile after taking into account the test parameters and the cavity volume. A closed form analytical solution is always desired for the inverse problem since it allows an easy implementation of the over-deterministic approach [23]. Unlike the case of pure molecular flow, the conductance in the transition regime is a function of the average pressure, which changes with time. As a result, a general simple closed form solution that defines the relationship between the apparent and true leak rates does not exist.

It is important to recall that viscous conduction dominates only when the leak channel opening (the true leak rate) is large and/or the average pressure is high. When the viscous contribution is high, helium leaks out fast during the preprocessing time. As a result, the internal pressure, and thus the pressure differential, drops so fast that the effect of the viscous conduction becomes insignificant after the preprocessing time. In other words, even when the viscous conduction is high after bombing, the contribution of viscous conduction decreases rapidly and the flow can be assumed molecular during the *measurement phase*, which provides a technical rationale for using the governing equations of molecular conduction to model the helium flow during the measurement phase.

The apparent leak rate can be modeled as:

(16) $$R(t)=\Omega e^{-\frac{L_{a}\;t}{{\mathrm{VP}}_{0}}{\left(\frac{M_{a}}{M_{\mathrm{helium}}}\right)}^{\frac{1}{2}}}=\Omega e^{-\frac{2.68L_{a}\;t}{{\mathrm{VP}}_{0}}}$$

where *M_a_* (28.7) and *M_helium_* (4) are the molecular weight of air and helium (in grams), respectively, and Ω is the apparent leak rate at the beginning of the measurement phase. By taking logarithms, Equation (16) can be written as:

(17) $$\text{ln}\;R(t)=\text{ln}\;\Omega -\left(\frac{2.68L_{a}}{{\mathrm{VP}}_{0}}\right)t$$

Under idealized conditions, the two unknowns (Ω and *L_a_*), can be obtained using two arbitrary data points in the apparent leak rate profile. In practice, however, the errors contained in the experimental data are not always negligible and this is the rationale of the least-squares approach to fit the experimentally determined data to the theoretical solution [23,24].

The least-squares method has been used in a regression analysis. The basic assumption that underlies this approach is that there are always differences between experimental results and theoretical values. Their relationship can be expressed using the error function, ϒ, as:

(18) $$ϒ={\sum_{k=1}^{n}\left[\text{ln}\;R_{t_{k}}-\left\{\text{ln}\;\Omega -\left(\frac{2.68L_{a}}{{\mathrm{VP}}_{0}}\right)\;t_{k}\right\}\right]}^{2}$$

where *n* is the number of data points, *R*(*t_k_*) and *t_k_* are the corresponding data points of the apparent leak rate profile. The objective is to find the values of Ω and *L_a_* that minimize the error function. This is achieved when the following conditions are satisfied:

(19) $$\frac{\partial ϒ}{\partial \Omega }=0\;\;\;\text{and}\;\;\;\frac{\partial ϒ}{\partial L_{a}}=0$$

Equation (19) can be solved numerically to determine *R_i_* and *L_a_*.

The method was implemented for a MEMS package. The package enclosed MEMS devices and comprised of a silicon cap bonded to a silicon substrate by means of a metallic seal. The overall package dimensions are 2.5 mm × 2.5 mm × 0.7 mm. The internal cavity volume, *V*, of the tested packages is 2.156 × 10^−4^ cm^3^.

**Figure 4.**
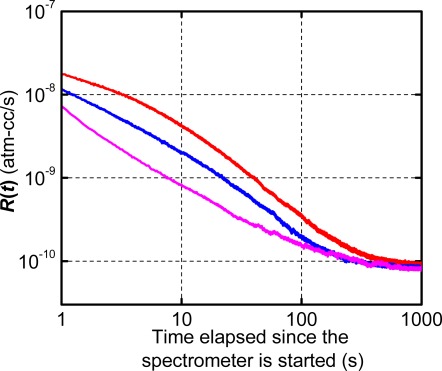
Representative zero signals.

The zero signal is a noise signal and should be excluded when the true leak rate is to be measured. Although it can vary slightly from an instrument to an instrument, the zero signal time can be measured experimentally simply by operating the mass spectrometer without any specimen inside the test chamber. Representative zero signals are shown in Figure 4. Although the initial signal strength varies, the signal stabilizes at a value of ∼ 10^−10^ atm-cm^3^/s after ∼150 s.

The following procedure was used in the experiment:

- A single package was subjected to pressurized helium (*P_b_* = 5 atm) for the duration of the bombing time, *t_b_* = 6 hours.
- It was transferred into the spectrometer in time, *t_dwell_*, of 10 minutes, and the spectrometer was switched on immediately after the dwell time.
- Data recording started after the zero signal time *t_zero_*, of 2.5 minutes.

**Figure 5.**
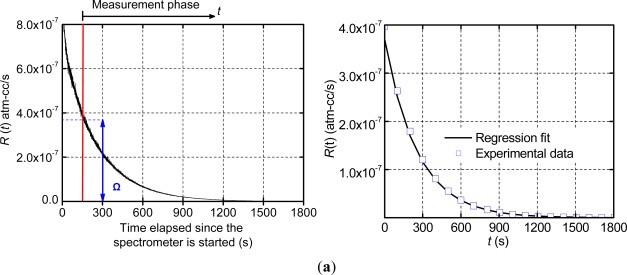

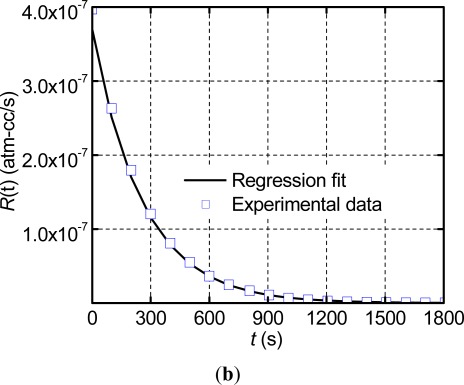
(**a**) Apparent leak rates of Package 1 obtained from the helium mass spectrometer the zero signal time and the apparent leak rate at the beginning of the measurement phase (Ω) are illustrated; and (**b**) the data of the measurement phase are repotted with the results from the regression analysis.

The apparent leak rate signal was measured using a commercial helium fine leak tester (Model DGC 1001, Alcatel). The data was recorded at 5 Hz and the results of Package 1 are shown in Figure 5(a), where the zero signal time and the apparent leak rate at the beginning of the measurement phase (Ω) are also illustrated.

The data of the measurement phase was utilized to determine the true leak rate through the regression analysis. The data was trimmed at a value of *R*(*t*) =2.5 × 10^−10^ atm-cm^3^/s in order to negate the effect of the stabilized zero signal (10^−10^ atm-cm^3^/s) on the regression. The analysis was conducted using MATLAB, and yielded a true leak rate value of 3.12 × 10^−7^ atm-cm^3^/s with the goodness of fit, R^2^, equal to 0.995. The experimental data of the measurement phase are replotted in Figure 5(b) together with the numerical result from the regressions analysis (*i.e.*, a plot of Equation (16) using the values of Ω and *L_a_* determined from the regression analysis). As expected from the extremely high value of R^2^, the regression results and the experimental data are nearly identical. It is to be noted that only a few experimental data points are shown in Figure 5(b) in order to distinguish them from the regression fit.

**Figure 6.**
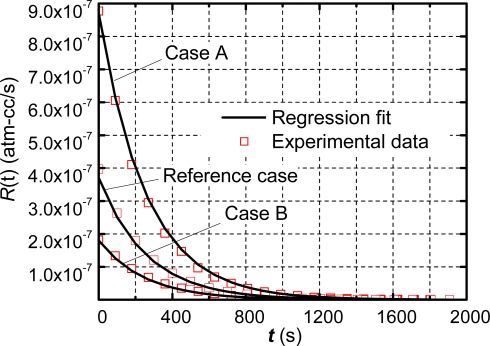
Apparent leak rates and the corresponding regression fits of Package 1 with various dwell times: Case A = 5 min; Case B = 20 min. The reference case has a dwell time of 10 min (Figure 5(b)).

The robustness of the technique was assessed by testing Package 1 again with different dwell times: 5 minutes (Case A) and 20 minutes (Case B). The apparent leak rate profile of each case and the corresponding regression fits are shown in Figure 6, where the reference case with a dwell time of 10 min is also shown for comparison.

The regression technique yields the true leak rate values of 2.99 × 10^−7^ atm-cm^3^/s (Case A) and 3.20 × 10^−7^ atm-cm^3^/s (Case B), which have less than 4% variation compared with the value of the reference case (3.12 × 10^−7^ atm-cm^3^/s). These consistent values validate the efficacy of the proposed method.

The value of Ω was treated as an unknown in the regression analysis. It is tempting to utilize the experimentally measured value of Ω to reduce the number of unknowns in Equation (16). A supplementary analysis was conducted to investigate the stability (convergence as well as accuracy) of the true leak rate solution with the experimentally measured value of Ω. The results revealed that the experimentally determined value of Ω did not alter the true leak rate significantly. This fact was attributed to the large number of data used in the *over-deterministic* approach in the current study.

In practice, the experimental value of Ω inherently contains uncertainties associated with the instrument, in particular, Helium mass spectrometer, and can be very unstable. If large, the uncertainties in Ω can affect the true leak rate, and it is suggested that the value of Ω treated as an unknown as proposed in this study.

## 4. True Leakage Measurement Polymer-Sealed Package [14]

An effective numerical scheme is developed to solve the governing equations (Equations (9) and (10)). The scheme is verified experimentally using the optical leak test.

### 4.1. Effective Volume Scheme

**Figure 7.**
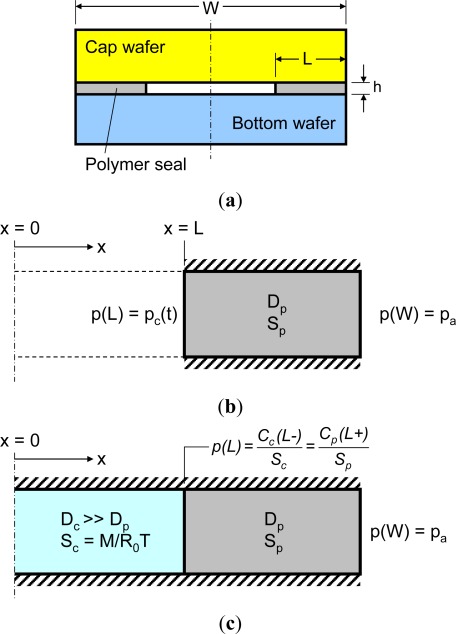
Schematic illustration of (**a**) the cross-section of the actual package, (**b**) the two-dimensional model and (**c**) the two-dimensional model with the “effective volume”.

As illustrated in Figure 7, a real 3-D package (Figure 7(a)) can be modeled as a 2-D structure (Figure 7(b)) since a cavity and a seal are sandwiched by an inorganic substrate or a silicon chip through which gas cannot penetrate or, if any, the amount is negligible. The 2-D diffusion model can be solved numerically using commercially available finite element analysis (FEA) software packages using the initial and boundary conditions defined in Equation (11). It is important to recall that the boundary condition at the polymer seal and cavity interface is transient; the cavity pressure increment at each time step should be calculated and subsequently used to update the boundary condition at the inner surface after each time step. This updating procedure requires a user-defined algorithm [15].

An effective modeling scheme is proposed to avoid the user-defined algorithm (this scheme will be referred to as “effective volume”). A schematic illustration of the effective volume scheme is shown in Figure 7(c). It models the package cavity as an imaginary polymer with an extremely large diffusivity and an “equivalent solubility”. The large diffusivity (several orders higher than that of the polymeric seals) ensures that the gas pressure is uniform within the cavity. It is important to note, however, that the solubility of the imaginary polymer cannot be chosen arbitrary. Instead the effective solubility should be derived from the gas law and Henry’s law as:

(20) $$S_{c}=\frac{C}{p}=\frac{\rho }{{\mathrm{nR}}_{0}\;T/V}=\frac{M}{R_{0}\;T}$$

where *ρ* is the gas density, which has the same dimension as the gas concentration (kg/m^3^; note that gas density can be interpreted as gas concentration inside the imaginary polymer), *V* is the gas volume (m^3^) and *n* is the number of moles (mol).

The effective volume scheme transforms the original single material diffusion problem with transient boundary conditions into a bi-material gas diffusion problem with fixed boundary conditions. Consequently, the Nernst distribution law should be considered for mass continuity at the cavity-polymer seal interface [the inner surface of the polymer seal, *x* = *L* in Figure 7(c)], which can be expressed as [15]:

(21) $$p(L)=\frac{C_{c}\;(L-)}{S_{c}}=\frac{C_{p}\;(L+)}{S_{p}}$$

where *C_c_* and *C_p_* are the gas concentration (density) of the cavity and the polymer seal, respectively, and *L* − and *L*+ are the (identical) *x* coordinate at the interface approached from the cavity side and from the seal side, respectively.

The effective volume modeling scheme can be readily implemented using commercial finite element analysis (FEA) software packages. Not all commercial FEA software packages offer the mass diffusion analysis function, but the current problem—namely, a diffusion analysis of a multi-material system subjected to an isothermal condition—can be solved by the thermal diffusion (or heat transfer) analysis function adopting the well-established thermal-moisture diffusion analogy [15,16]. With both analysis functions, the mass continuity (including the Nernst distribution law defined in Equation (21)) is automatically satisfied at the interface and the user-defined algorithm is not required. The accuracy of the proposed effective-volume scheme has been confirmed by a direct numerical solution of Equation (6) using the finite difference method (FDM) [15].

### 4.2. Optical Leak Test

**Figure 8.**
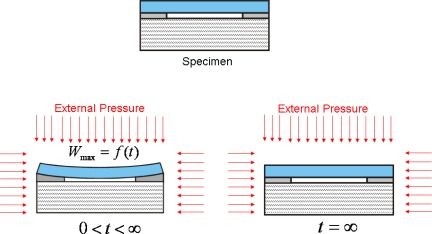
Schematic illustration of the optical leak test.

The basic principle of the optical leak test [11,25,26] is depicted in Figure 8. A MEMS package is first subjected to a pressurized gas (*i.e.*, constant external pressure). As gas leaks into the package, the pressure differential (*i.e.*, the difference between the external pressure and the cavity internal pressure) changes over a period of time. This change in pressure differential induces a change in specimen deformation that is recorded experimentally as a function of time. The experimental data is converted to the pressure differential using the pre-determined relationship between the pressure differential and the specimen deformation (calibration curve). Then, the time-dependent internal cavity pressure can be determined by subtracting the pressure differential from the known external pressure.

#### Experimental Setup

**Figure 9.**
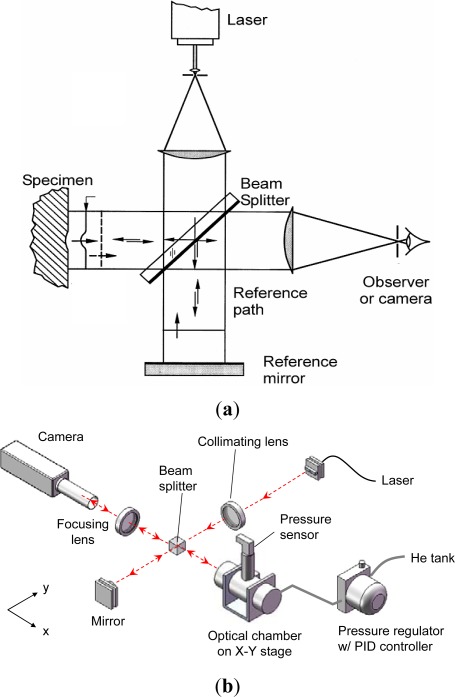
Schematic illustration of (**a**) Twyman-Green interferometry and (**b**) the experimental setup.

The optical/mechanical configuration is shown schematically in Figure 9. The specimen is held inside a cylindrical stainless steel pressure chamber, which is provided with a window for direct viewing. The pressure vessel is mounted on a heavy duty stage in order to prevent vibrations on account of forces exerted by the pressure tubing that supplies gas into the vessel. This stage offers x-y translation and rotational adjustment of the vessel, and hence the specimen inside it as desired. The gas pressure is regulated by a PID controller (TESCOM ER3000).

The surface topology of the package is documented by a classical laser interferometry configuration called Twyman/Green interferometry [27]. The technique is simple and is ideally suited for MEMS packages since the package surface is specular, which is a critical requirement for the method. As illustrated in Figure 9(a), an expanded laser beam is collimated by a collimating lens. The collimated light is split into two—one directed towards the specimen and the other towards the reference mirror (an optical flat). The reflected wave fronts recombine and interfere to form an interferogram (or fringe pattern). The interferogram provides a contour map of the surface topography.

The optical/mechanical configuration of the actual experimental setup is illustrated in Figure 9(b). There are two major parts in the setup: (1) an optical setup for deformation measurement and (2) a pressure chamber with a high-precision pressure regulation system. The specimen is held inside a cylindrical stainless steel pressure vessel, which is provided with a window for direct viewing. Both the vessel and the window are designed to withstand pressures up to 50 atm. The pressure vessel is mounted on a heavy duty stage to prevent vibrations caused by the vessel’s gas supply tubing. This stage offers x-y translation and rotational adjustment of the vessel, and hence the specimen inside it as desired. The fringe pattern is captured by a high resolution camera with a 1″ CCD format (Pulnix TM-1040) through an imaging lens.

Any high pressure gas tank can be used as the source of gas. A mechanical regulator located on the tank reduces the gas pressure from the tank pressure value (∼70 atm) to 7 atm. This lower pressure gas is then supplied to a PID controller (TESCOM ER3000). The PID controller has an internal sensor, which is used in conjunction with PID logic and user defined PID parameters to reduce gas pressure to the desired pressurization value. An additional pressure sensor (TESCOM 200-1000-2527) is screwed into the pressure vessel in order to read the pressure inside the chamber, which can detect any large leakage of gas due to an accidental failure/rupture of the chamber gaskets and seals. The uncertainty of measurement using this pressure regulation setup is ±0.02 atm (±0.3 psi).

The deformation, *W*(*x,y*), at any point on the specimen is given by:

(22) $$W(x,\;y)=\frac{\lambda }{2}\;N(x,\;y)$$

where *N*(*x,y*) is the fringe order at a point (*x,y*) and *λ* is the wavelength of the laser. The basic contour interval of this arrangement is defined as *λ*/2. When a helium neon laser (*λ* = 632.9 nm) is employed in the setup, it provides the basic contour interval of 316.5 nm/fringe order.

The fringe patterns are processed further by the Fast Fourier Transform (FFT) method [28,29] to enhance the displacement resolution. The FFT method was utilized for an automatic fringe analysis since the region of interest does not contain any boundaries and the deformation of the package surface varies smoothly. An added benefit of the FFT method is that the inherent high frequency random noise can be eliminated effectively during the inverse FFT process. The FFT method is illustrated using the actual package below. A more detailed mathematical description of the method can be found in [28].

**Figure 10.**
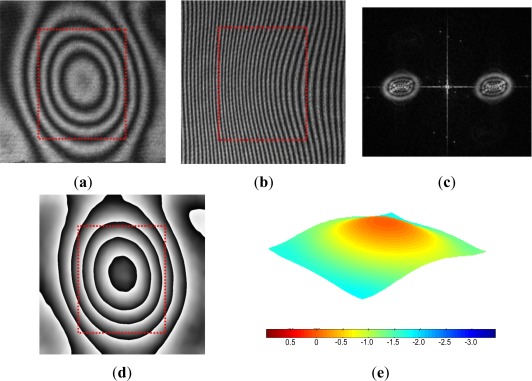
Illustration of FFT analysis: (**a**) Original fringe pattern, (**b**) Modulated pattern with carrier fringes, (**c**) Fourier spectra, (**d**) Phase map after inverse Fourier transform and (**e**) 3-D plot. The cavity location is indicated by the dotted box.

The original fringe pattern of the specimen before pressurization is shown in Figure 10(a). A carrier pattern of constant displacement gradient is added to the original pattern by a small rigid body rotation of the specimen; the number of carrier fringes over the cavity was about 20 which was equivalent to 6.3 μm. The modulated pattern is shown in Figure 10(b). After the two-dimensional FFT, the real harmonic is isolated in the frequency domain [Figure 10(c)]. The center of the spectrum is moved to the origin of the frequency axis to remove the carrier frequency in the frequency domain. Then, the inverse Fourier transform is performed to restore the original phase map [Figure 10(d)]. Unwrapping of this phase map yields a fractional fringe orders with high fidelity at every point. This information is used to generate a 3D deformation map shown in Figure 10(e). The configuration used in the experiment offered the displacement measurement resolution of ±15 nm and the pressure regulation accuracy of ±0.3 psi (±0.02 atm).

### 4.3. Experimental Validation

The package used in the experiment consists of a glass cap bonded to a silicon substrate using a photo-definable adhesive polymer. The cavity was fabricated through a lithography process. All the processes were conducted in a controlled nitrogen environment (0.9 bar). The height of the silicon substrate, the glass cap and the polymer seal are 120 μm, 500 μm and 46 μm, respectively. The overall package dimensions are 4.6 mm × 4.5 mm. The cavity dimensions are 2.22 mm × 2.86 mm, which yields an internal cavity volume of ∼ 3 × 10^−4^ cm^3^.

**Figure 11.**
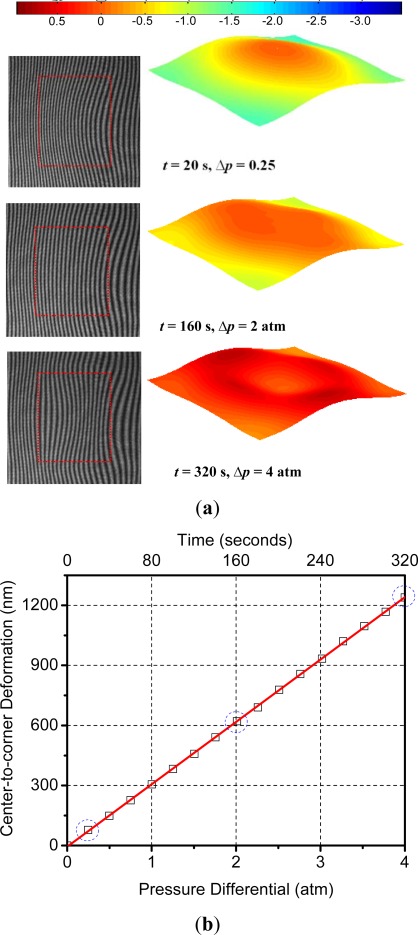
(**a**) Representative fringe patterns and 3-D deformation maps obtained during the calibration process (the units in the scale are μm and the cavity location is indicated by the dotted box); (**b**) calibration curve (the encircled values correspond to the fringes in (a).

In order to obtain the calibration curve, the pressure in the chamber was increased to 4 atm (gauge) in steps of 0.25 atm and the surface deformation was recorded at each step. The deformation-induced deflections are plotted as a function of the applied external pressure and bombing time in Figure 11(a). Three data points marked by a dotted circle were obtained from the representative fringe patterns and the corresponding 3-D maps shown in Figure 11(b). From this plot the following linear relationship between pressure differential, *Δp,* and the maximum deflection, *W_max_*, of the specimen was obtained:

(23) $$W_{\text{max}}=309.58\;(\Delta p)$$

where the units for pressure and deformation are atm and nm, respectively. It is worth noting that the above relationship is valid for both bombing and release stages since the deformation is within the elastic range. This was confirmed again in the actual measurement for cavity pressure evolution.

**Figure 12.**
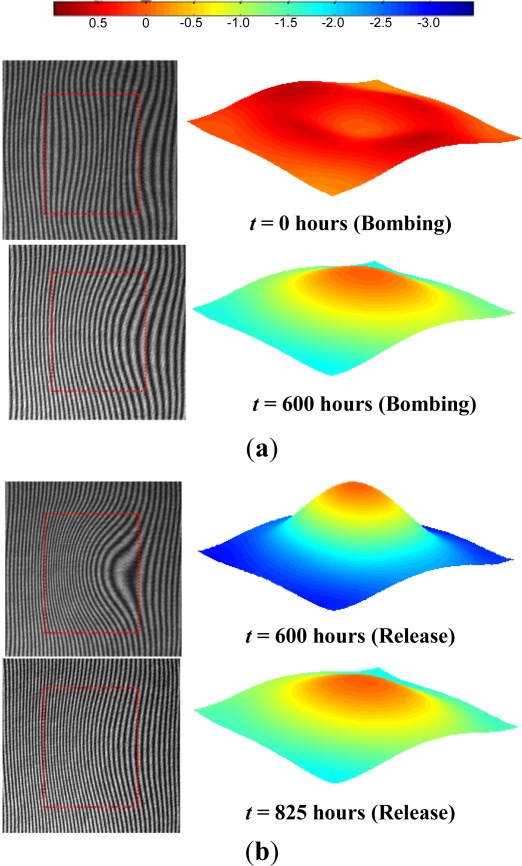
Cap surface topographical contour maps (the units in the scale are μm and the cavity location is indicated by the dotted box) at the beginning and the end of (**a**) bombing stage and (**b**) release stage.

After the calibration curve was obtained, the package was subjected to a constant bombing pressure of 4 atm (gage) and the deflections were measured as a function of time. The bombing pressure was maintained for 600 h. There was no noticeable deflection change after 600 h, indicating that the cavity pressure was equal to the bombing pressure. At this point, the “release” stage was initiated by closing the helium gas valve and opening the chamber to the atmospheric environment (0 atm of helium). The surface deflection was also documented regularly during the release stage. Representative fringe images and corresponding 3D maps are shown in Figure 12(a,b) for the bombing and release stages, respectively.

**Figure 13.**
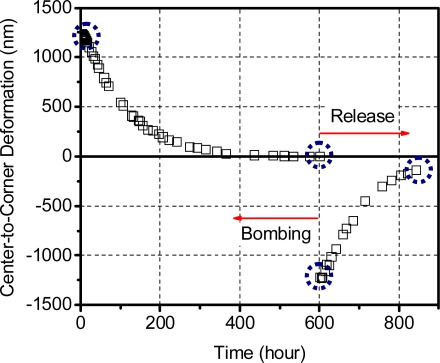
Effective chip surface deflections during the bombing and release stages; the encircled values correspond to the contour maps shown in Figure 12.

**Figure 14.**
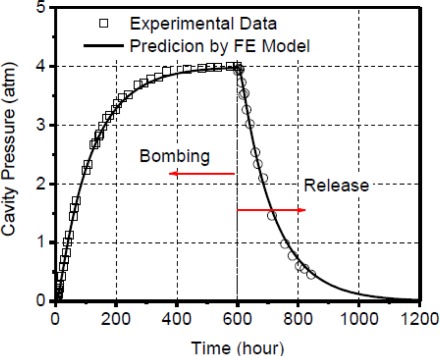
Cavity pressure evolutions measured by the optical leak test are compared with the numerical predictions.

The effective deflections obtained from the 3-D maps are plotted in Figure 13, where the data points marked by a dotted circle were obtained from the results shown in Figure 12. Using the calibration curve (Equation (23)), the deflection values during the bombing and release stages were converted into pressure differential values. The internal cavity pressure was then calculated by subtracting these values from the known external pressure (4 atm while bombing and 0 atm during release) and is plotted in Figure 14.

A finite element model based on the effective-volume scheme was built to simulate the cavity pressure evolution during the bombing and release stage. The modeling prediction is compared with the experimental data in Figure 14. It is evident that the diffusion model follows the experimentally observed cavity pressure change extremely accurately. This corroborates the validity of the diffusion based hermetic behavior of polymer-sealed MEMS packages and also validates the assumptions used in the boundary conditions for the finite element model.

It is to be noted that the two diffusion properties (diffusivity and solubility) required for the modeling were not known in advance. Instead they were determined through an inverse analysis [23,24]. The goal of the analysis is to find the *D-S* combination that produces the most accurate cavity pressure prediction. The details of the approach to determine the diffusion constants can be also found in reference [15].

## 5. Summary

Two distinctively different techniques were reviewed to measure true leak rates of MEMS packages with different sealing materials: a Helium mass spectrometer based technique for metallic sealing and a gas diffusion based model for polymeric sealing. The governing equations were reviewed and the measurement procedures were discussed. The true leak rates of MEMS packages with micro to nanoliter cavity volumes can be measured accurately by combining the two techniques.
